# Rap1, Canoe and Mbt cooperate with Bazooka to promote zonula adherens assembly in the fly photoreceptor

**DOI:** 10.1242/jcs.207779

**Published:** 2018-03-15

**Authors:** Rhian F. Walther, Mubarik Burki, Noelia Pinal, Clare Rogerson, Franck Pichaud

**Affiliations:** MRC Laboratory for Molecular Cell Biology, University College London, Gower Street, London WC1E 6BT, UK

**Keywords:** Epithelial polarity, Pak4, Par3, AFDN, Bazooka, Photoreceptor, Rap1, Zonula adherens

## Abstract

In *Drosophila* epithelial cells, apical exclusion of Bazooka (the *Drosophila* Par3 protein) defines the position of the zonula adherens (ZA), which demarcates the apical and lateral membrane and allows cells to assemble into sheets. Here, we show that the small GTPase Rap1, its effector Canoe (Cno) and the Cdc42 effector kinase Mushroom bodies tiny (Mbt), converge in regulating epithelial morphogenesis by coupling stabilization of the adherens junction (AJ) protein E-Cadherin and Bazooka retention at the ZA. Furthermore, our results show that the localization of Rap1, Cno and Mbt at the ZA is interdependent, indicating that their functions during ZA morphogenesis are interlinked. In this context, we find the Rap1-GEF Dizzy is enriched at the ZA and our results suggest that it promotes Rap1 activity during ZA morphogenesis. Altogether, we propose the Dizzy, Rap1 and Cno pathway and Mbt converge in regulating the interface between Bazooka and AJ material to promote ZA morphogenesis.

## Abstract (precis)

**Summary:** Converging pathways regulate adherens junction stability and retention of Bazooka to promote zonula adherens maturation during epithelial cell morphogenesis in *Drosophila*.

## INTRODUCTION

The epithelial zonula adherens (ZA) enables cell–cell adhesion, allowing epithelial cells to assemble into sheets and form organs. Elucidating how ZA morphogenesis is regulated during epithelial cell morphogenesis remains an important goal in epithelial cell biology. The ZA includes the adhesion molecule E-Cadherin (E-Cad; known as Shotgun in flies) and its effector β-catenin [known as Armadillo (Arm) in flies], which are the main adherens junction (AJ) components that mediate cell–cell adhesion (Tepass, 2012). Several factors regulate the morphogenesis and accumulation of AJ material during ZA assembly. These include the small GTPase Rap1 and its effector actin-binding protein Canoe (Cno; the homolog of mammalian AF6, also known as AFDN) (Bos et al., 2001; Niessen and Gottardi, 2008; Pannekoek et al., 2009), the type 2 p21-activated kinase Mushroom bodies tiny (Mbt, the homolog of Pak4) (Menzel et al., 2007; Wallace et al., 2010; Walther et al., 2016) and the Par complex (Cdc42–Par6–aPKC–Bazooka) (McGill et al., 2009; Morais-de-Sá et al., 2010; Walther and Pichaud, 2010). However, we lack an integrated view of how these factors come together to regulate ZA morphogenesis and remodeling during epithelial cell morphogenesis.

**Fig. 1.**
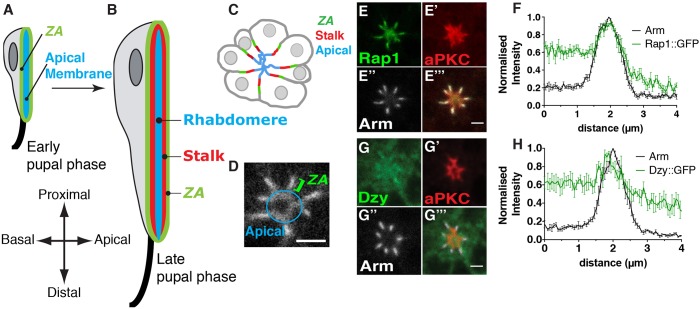
**Dizzy and Rap1 are ZA-associated proteins.** (A,B) Schematic representation of the developing pupal photoreceptor. (A) Early and (B) late stage pupal photoreceptors shown along the lens (top) to brain (bottom) axis of the retina. The apical membrane, which is clearly differentiated by mid pupation and by late pupation forms the rhabdomere, is depicted in blue. The stalk membranes are depicted in red and the ZA in green. The axon is depicted as a black line, at the bottom (brain or distal pole) of the cell. (C) Cross section of a cluster (ommatidium) of photoreceptors at mid pupation when the ZA (green), stalk membrane (red) and apical membrane (blue) have been specified. (D) Annotated magnification of the Rap1::GFP staining showing the apical membrane and the ZA. (E–E‴) Photoreceptors expressing Rap1::GFP (E), stained for aPKC (E′) and Arm (E″). Scale bar: 2 µm. (F) Intensity profiles of Rap1::GFP and Arm measured along the apical-basal axis. Results are mean±s.e.m. (*n*=8 cells from 3 retinas). (G–G‴) Photoreceptors expressing Dzy::GFP (G), stained for aPKC (G′) and Arm (G″). Scale bar: 1.5 µm. (H) Intensity profiles of Dzy::GFP and Arm measured along the apical-basal axis. Results are mean±s.e.m. (*n*=6 cells from 3 retinas).

The *Drosophila* pupal photoreceptor has long been used as a model system to study the genetic and molecular basis of the specification and morphogenesis of the epithelial apical, subapical and ZA membrane domains. In these cells, these domains are clearly separated along the apical basal (*x*–*y*) axis (Fig. 1A–C), and the apical organelle, called the rhabdomere, is analogous to the epithelial brush border and consists of ∼60,000 microvilli. The subapical membrane is called the stalk and can be up to 1.5 µm in length, and connects the rhabdomere to the more basal ZA. These three membrane domains are specified early during pupal development and undergo sustained morphogenesis as the cells elongate by ∼10-fold to generate the lens (proximal) to brain (distal) axis of the retina (Ready, 2002) (Fig. 1A,B). In pupal photoreceptors, the Par complex regulates the separation of the ZA from the stalk membrane (Hong et al., 2003; Nam and Choi, 2003; Walther et al., 2016; Walther and Pichaud, 2010). Concomitantly, the conserved transmembrane protein Crumbs (Crb) functions with the Par complex to drive stalk membrane and ZA morphogenesis as photoreceptors elongate along the proximal-distal axis of the retina (Izaddoost et al., 2002; Pellikka et al., 2002).

In *Drosophila* epithelia, Bazooka (Baz) phosphorylation at serine S980 (P-S980-Baz) by atypical protein kinase C (aPKC) is essential for specifying the ZA and subapical membrane. Baz phosphorylation occurs upon Par complex assembly and is thought to allow for Crb to capture the Cdc42–Par6–aPKC complex, thus leading to the apical exclusion of P-S980-Baz (Krahn et al., 2010; Morais-de-Sá et al., 2010; Walther and Pichaud, 2010). Confined to the apical-lateral border of the cell, P-S980-Baz is then thought to promote ZA assembly, at least in part through its ability to bind to Arm (Wei et al., 2005). In the pupal photoreceptor, Crb and Par6–aPKC accumulate at the stalk membrane and P-S980-Baz is found immediately basal to it, at the developing ZA (Fig. 1B,C). It is likely that Par3 phosphorylation and its concomitant apical exclusion play a similar role in vertebrate neuroepithelial cells. In vertebrates, Par3 is phosphorylated by aPKC (Nagai-Tamai et al., 2002), and in neuroepithelial cells, is found basal to aPKC and Par6, at the apical junctional complex (AJC)*,* which contains cadherins (Aaku-Saraste et al., 1996; Afonso and Henrique, 2006).

In addition to Baz, the p21-activated kinase Mushroom bodies tiny (Mbt) and its vertebrate homolog Pak4 have also been shown to regulate ZA morphogenesis. In pupal photoreceptors, Mbt regulates ZA morphogenesis and overall apical membrane differentiation by promoting the accumulation of the E-Cad–Arm complex via phosphorylating Arm and regulating the F-actin cytoskeleton, which in turn is essential for the retention of Baz at the ZA (Jin et al., 2015; Law and Sargent, 2014; Menzel et al., 2008; Schneeberger and Raabe, 2003; Walther et al., 2016). In these cells, failure to retain AJ material, including Baz at the ZA, leads to a shortening of the ZA along the apical-basal axis of the cell. In addition, severe defects in polarized photoreceptor morphogenesis can occur (Walther et al., 2016). In vertebrate cells, Pak4 also regulates ZA maturation (Jin et al., 2015; Law and Sargent, 2014; Wallace et al., 2010), and its function during epithelial morphogenesis has been linked to that of the Par complex, as Pak4 phosphorylates Par6b (Jin et al., 2015; Wallace et al., 2010). While in flies Mbt does not phosphorylate Par6, Mbt and Baz are the main regulators of AJ material accumulation at the plasma membrane*.* In the absence of *baz*, AJ material can still be detected at the plasma membrane of pupal photoreceptors within the apical pole of the cell. Similarly, ZA domains are present in *mbt-*null mutant cells, although they are shorter and present less AJ material than in wild-type cells*.* However, no AJ domains are found in photoreceptors mutant for both *baz* and *mbt*, indicating that Baz and Mbt function in parallel pathways to promote AJ morphogenesis and/or stabilization at the plasma membrane (Walther et al., 2016).

Another conserved factor that regulates AJ material morphogenesis is Rap1, which in epithelia can be activated by the PDZ-containing guanine exchange factor (GEF) protein Dizzy (Dzy) (de Rooij et al., 1999; KawAJiri et al., 2000). Rap1 has been shown to localize at the AJ in various fly epithelia, and to be an essential AJ regulator (Boettner et al., 2003; Boettner and Van Aelst, 2007; Choi et al., 2013; Knox and Brown, 2002; O'Keefe et al., 2009; Spahn et al., 2012; Wang et al., 2013). In the fly embryo, Rap1 and its effector F-actin-binding protein Cno (Boettner et al., 2003; Mandai et al., 2013; Sawyer et al., 2009) regulate the apical localization of both Baz and Arm, with Baz reciprocally influencing Cno localization. Furthermore, Baz is required to capture preassembled AJ material, thus promoting the morphogenesis of spot AJ*s*, which are precursors of the ZA in this tissue (McGill et al., 2009). In addition, work in human MCF7 cells has shown a role for Rap1 (which has two forms, Rap1a and Rap1b) during AJ maturation via promoting E-Cad recruitment at the sites of cell–cell contact, a function that has been shown to be mediated, at least in part, by Cdc42 (Hogan et al., 2004). However, how the functions of Rap1, Cno, Baz and Mbt relate to each other during ZA morphogenesis has not been examined. Here, we used the *Drosophila* photoreceptor system to investigate these relationships.

## RESULTS

### Rap1 regulates pupal photoreceptor ZA morphogenesis

In the fly retina, Rap1 has been previously shown to regulate AJ remodeling between newly specified photoreceptors, and between retinal accessory cells that surround the photoreceptors (cone and pigment cells) (O'Keefe et al., 2009). To examine the distribution of Rap1 and its GEF Dzy in the pupal photoreceptor (Fig. 1A–C), we made use of the *rap1-Rap1::GFP* and *dzy-Dzy::GFP* transgenes, which allow for expression of these proteins under the control of their endogenous promoter. We found that Rap1::GFP is present at the apical membrane and accumulates predominantly at the developing ZA (Fig. 1D–F). Dzy::GFP (Fig. 1G) shows a low level expression all over the apical membrane and presents a slight but reproducible enrichment at the developing ZA (Fig. 1G,H). These results suggest that Dzy and Rap1 might regulate apical membrane and ZA morphogenesis in the pupal photoreceptor.

**Fig. 2.**
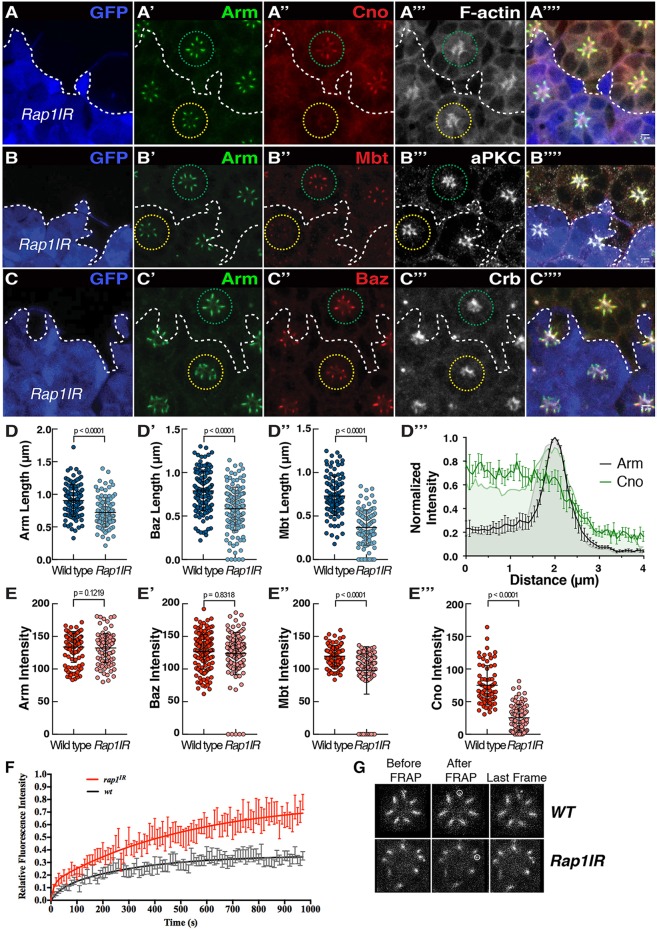
**Rap1 regulates the accumulation of AJ material during ZA morphogenesis.** (A–C) *Rap1IR* cells positively labeled for GFP (blue, the edge of which is denoted by the dashed line) and stained for Arm (A′,B′,C′), Cno (A″), Mbt (B″), Baz (C″), F-actin (A‴), aPKC (B‴) and Crb (C‴). Green circle, outline of wild-type ommatidia; yellow circle, outline of *Rap1IR* ommatidia. Scale bars: 2 μm. (D–D″) Quantification of Arm (D), Mbt (D′), Baz (D″) domain length at the ZA. Results are mean±s.d. (*n*≥105). (D‴) Normalized intensity profiles of Cno (green) and Arm (gray) in WT photoreceptors (shaded profiles) and *Rap1IR* photoreceptors. Results are mean±s.e.m. (*n*=7 cells from 3 retinas). (E–E‴) Quantification of Arm (E), Mbt (E′), Baz (E″) and Cno (E‴) mean pixel intensity at the ZA. Results are mean±s.d. (*n*≥105). (F) FRAP curve fit for E-Cad::GFP in wild-type (black) and *Rap1IR* (red) photoreceptors. For both genotypes, the basal end of the developing ZA (dashed circle) was photo-bleached (G). For wild-type ZA FRAP, *n*=14 and for *Rap1IR, n*=12. Error bars are s.e.m.

To assess the function of Rap1 during photoreceptor morphogenesis, we made use of available *Rap1* loss-of-function alleles. We found that generating mutant clones using the strong allele *Rap1^CD3^*, or expressing high levels of a previously validated *Rap1IR* (*Rap1* RNAi) construct (O'Keefe et al., 2009), leads to severe defects in recruiting the full complement of retinal accessory cells including the cone cells (Fig. S1A). Missing cone and pigment cells lead to retinal cell delamination, with many photoreceptors found below the floor of the retina (Fig. S1B–D), preventing us from assessing polarity and ZA morphogenesis. In order to bypass this strong phenotype, we limited the expression of *Rap1IR*. Decreasing the expression of *Rap1* at pupal stages did not affect photoreceptor apical-basal polarity in the majority of ommatidia examined (Fig. 2A–C). However, quantification revealed that the length of the Arm, Mbt and Baz domains, measured along the apical-basal axis, were significantly reduced when compared to those in wild type (Fig. 2D–D″). In addition, while the levels of Arm and Baz were comparable to those measured in wild-type cells (Fig. 2E,E″), we found that Cno accumulation at the ZA was nearly abolished (Fig. 2A″,D‴,E‴) and Mbt levels were significantly decreased when compared to wild type (Fig. 2B″,E′). We also note that apical levels of F-actin (Fig. 2A‴), aPKC (Fig. 1B‴) and Crb (Fig. 2C‴), were not affected in *Rap1IR* photoreceptors when compared to those in wild type. These data indicate that Rap1 is required for the accumulation or retention of Cno and Mbt at the developing ZA and for regulating the length of the ZA along the apical-basal axis.

### Rap1 promotes E-Cadherin stabilization at the ZA

We have previously shown that, in pupal photoreceptors, loss of *mbt* function leads to an increase in the mobile fraction of E-Cad at the ZA when compared to wild type as measured over 250 s (Walther et al., 2016). Our analysis of *Rap1IR* indicates that Mbt accumulation is strongly reduced at the ZA (Fig. 2B″,E′), which should therefore be accompanied by an increase in E-Cad mobility. To assess whether this is the case, we made use of fluorescence recovery after photobleaching (FRAP) and compared the recovery of an *ubi-E-Cad::GFP* transgene in wild-type and *Rap1IR* photoreceptors. In wild-type cells, over ∼250 s, we estimated that 25% of E-Cad::GFP was mobile (data not shown), which is consistent with previous estimations from our laboratory (Walther et al., 2016). However, while E-Cad::GFP shows a stronger recovery over this relatively short time scale in *Rap1IR* cells than in wild-type cells, the GFP signal failed to plateau (data not shown), preventing us from extrapolating the mobile fraction. We therefore performed FRAP over a longer time scale (1000 s). Over this long time scale, we found that ∼35% of E-Cad::GFP was mobile in the wild-type ZA, while ∼70% was mobile in *Rap1IR* photoreceptors (Fig. 2F,G). These data indicate that Rap1 promotes E-Cad stabilization at the ZA*,* and are compatible with Mbt mediating part of the Rap1 function during this process.

### Dzy regulates ZA morphogenesis through Rap1

**Fig. 3.**
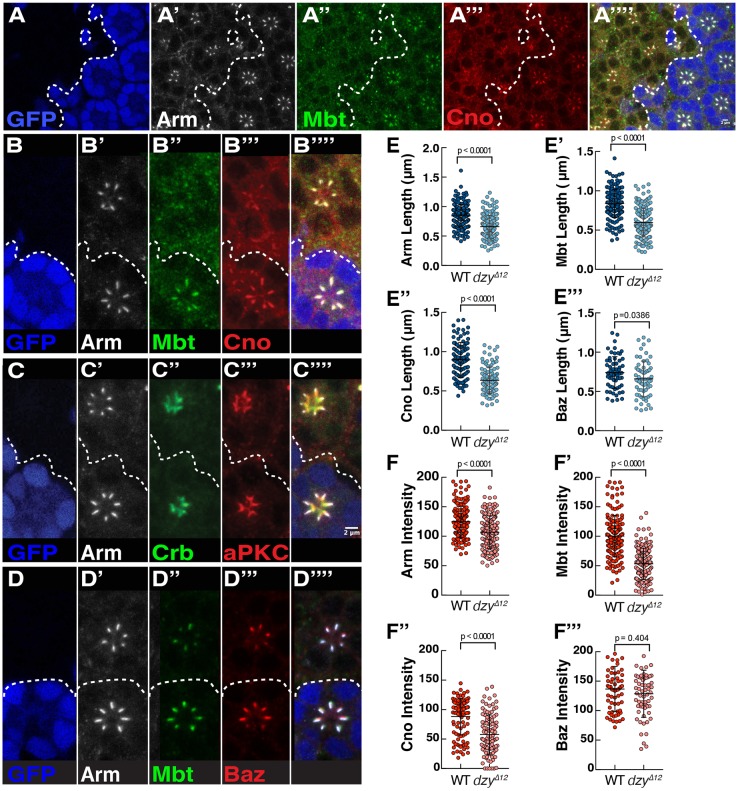
**Dzy regulates Cno and Mbt accumulation at the ZA*.*** (A–A‴) *dzy^Δ12^* mutant clone labeled by the lack of nuclear GFP (blue, the contour of which is denoted by the dashed line), stained for Arm (A′), Mbt (A″) and Cno (A″″). (B–B‴) An ommatidium mutant for *dzy* (lacking GFP, blue, B), stained for Arm (B′), Mbt, (B″) and Cno (B‴). (C–C‴′) Ommatidium mutant for *dzy* (lacking GFP, blue, C), stained for Arm (C′), Crb (C″) and aPKC (C‴). (D–D‴′) Ommatidium mutant for *dzy* (lacking GFP, blue, D), stained for Arm (D′), Mbt (D″) and Baz (D‴). Scale bars: 2 µm. (E–E‴) Quantification of Arm (E), Mbt (E′), Cno (E″) and Baz (E‴) domain length at the ZA. (F–F‴) Quantification of Arm (F), Mbt (F′), Cno (F″) and Baz (F‴) mean pixel intensity at the ZA. All error bars are s.d. (*n*≥70 from 4 retinas).

To examine the function of the Rap1-GEF *dzy* during photoreceptor morphogenesis, we made use of the strong *dzy^Δ12^* allele. We found that reducing *dzy* expression leads to a phenotype similar to that seen in the hypomorphic *Rap1IR* photoreceptors (Fig. 3A), including a notable decrease the length of the Arm, (Fig. 3A′,B′,C′,D′,E), Mbt (Fig. 3A″,B″,D″,E′), Cno (Fig. 3A‴,B‴,E″) and Baz (Fig. 3D‴,E‴) domains along the apical basal axis of the cell. In addition, the levels of Arm, Mbt and Cno are significantly reduced at the ZA when compared to those in wild-type cells (Fig. 3F–F″), but those of Baz were similar to that measured in wild-type cells (Fig. 3F‴). Consistent with Dzy acting as a Rap1-GEF in photoreceptors, removing a copy of the *dzy* locus enhances the mild rough-eye phenotype obtained when reducing the expression of *Rap1IR* (Fig. S2). However, we note that the *dzy* loss-of-function phenotype is much milder than that of *Rap1^CD3^* and that seen upon strong *Rap1IR*, in that no cells delaminate below the floor of the retina in *dzy* mutant clones. Other Rap1-GEFs must therefore be at play in the developing retina.

### Cno couples Arm and Baz at the ZA and is required for the apical accumulation of aPKC and Crb

**Fig. 4.**
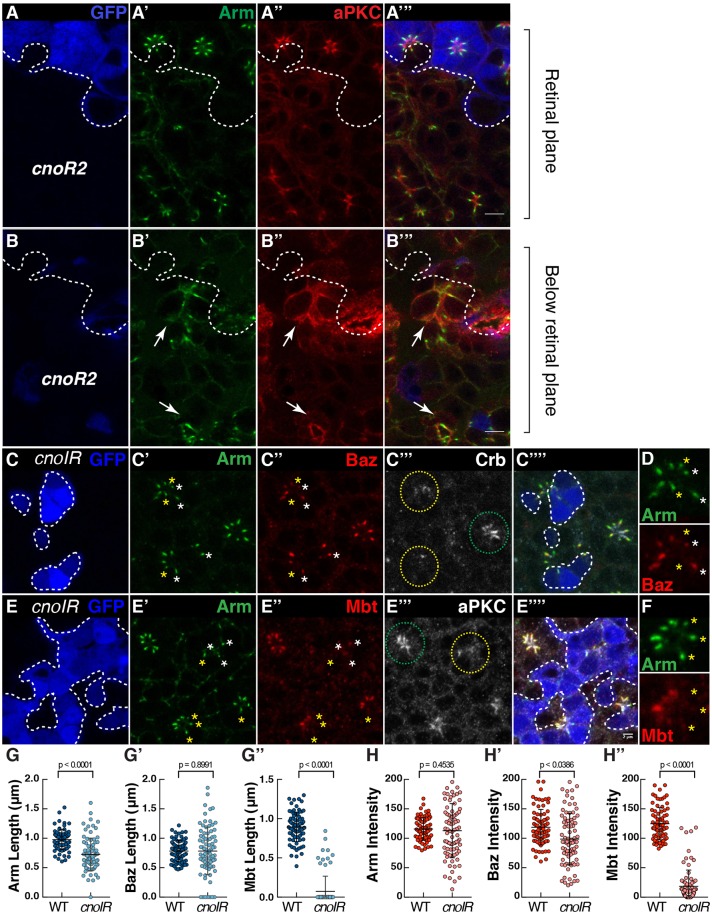
**Cno regulates the coupling of Arm, Baz and Mbt at the developing ZA*.*** (A–B) *cno^R2^* mutant cells (lacking GFP, blue, the contour of which is denoted by the dashed line, A,B) stained for Arm (A′,B′) and aPKC (A″,B″). White arrows indicate *cno^R2^* mutant photoreceptors that have delaminated from the retinal neuroepithelium. (C–F) *cnoIR* clones positively labeled by GFP (blue, C,E) and stained for Arm (C′,E′), Baz (C″), Crb (C‴), Mbt (E″) and aPKC (E‴). Green and yellow circles outline wild-type and *cnoIR* ommatidia, respectively. (D,F) A magnification of one mosaic ommatidium to highlight the absence of Baz (D) or Mbt (F) in some of the Arm domains. White stars label ZAs containing both Arm and Baz, while yellow stars indicate ZAs containing Arm but depleted for Baz (D) or containing Arm but depleted for Mbt (F). Scale bars: 2 μm. (G–G″) Quantification of Arm (G), Baz (G′) and Mbt (G″) domain length at the ZA. (H–H″) Quantification of Arm (H), Baz (H′) and Mbt (H″) mean pixel intensity at the ZA. All error bars are s.d. (*n*≥77 from 5 retinas).

As well as regulating Mbt accumulation at the ZA, one likely mechanism whereby Rap1 might promote E-Cad stabilization is through the F-actin linker Cno. In the pupal photoreceptor, Cno is enriched at the ZA and found at low levels at the apical membrane, which is similar to the Arm expression pattern (Fig. 2A″,D‴). To assess Cno function, we made use of the strong *cno^R2^* allele. We found that *cno^R2^* mutant photoreceptors delaminate through the floor of the retina (Fig. 4A,B), a phenotype resembling that obtained when strongly reducing *Rap1* expression. As with *Rap1^CD3^*, the polarity of the delaminated photoreceptors is strongly compromised in *cno^R2^* mutant cells, and the delamination phenotype is likely due to defects in assembling the full complement of interommatidial accessory cells. In order to circumvent the delamination phenotype, we made use of *cnoIR* (*cno* RNAi). Examining retinas mosaic for *cnoIR* revealed that Cno regulates the length of the ZA and is required for the accumulation of Arm (Fig. 4C′,4E′,G,H), Baz (Fig. 4C″,H′) and Mbt (Fig. 4E″,G″,H″) at the developing ZA. We also noted instances where Arm was present at the ZA but Mbt was absent (Fig. 4D,F). The similarity between the *Rap1IR* and *cnoIR* ZA phenotypes suggests that the function of Rap1 and Cno during ZA morphogenesis are linked. However, in the case of *cnoIR*, we also detect ZAs without Baz, a phenotype not detected in *Rap1IR* and indicative of a failure in retaining Baz at the developing ZA. Lack of Baz at the ZA is seen when overexpressing a version of Arm that cannot be phosphorylated by Mbt (ArmSA^mbt^) raising the possibility Mbt mediates Cno function (Walther et al., 2016). To test this possibility we expressed a phospho-mimetic version of Arm (ArmSE^mbt^) in *cnoIR* retinas. However, this did not ameliorate the *cnoIR* phenotype when considering ZA length along the apical-basal axis or Baz retention at the ZA (Fig. S3).

In addition, we observed that unlike for *Rap1IR*, levels of Crb and aPKC were decreased in *cnoIR* mutant cells (Fig. 4C‴,E‴), indicating that Cno might regulate the accumulation of these factors during apical membrane morphogenesis. However, we note that our manipulation of Rap1 levels using *Rap1IR* does not lead to a complete loss of Cno at the ZA (Fig. 2A″,E‴), while Cno is virtually undetectable in our *cnoIR* experiments (Fig. S4). We therefore envisage that residual Cno in *Rap1IR* is sufficient to support the retention of Baz at the ZA, and the apical accumulation of Crb and aPKC.

### Mbt is required for the accumulation of Cno and enrichment of Rap1 at the ZA

**Fig. 5.**
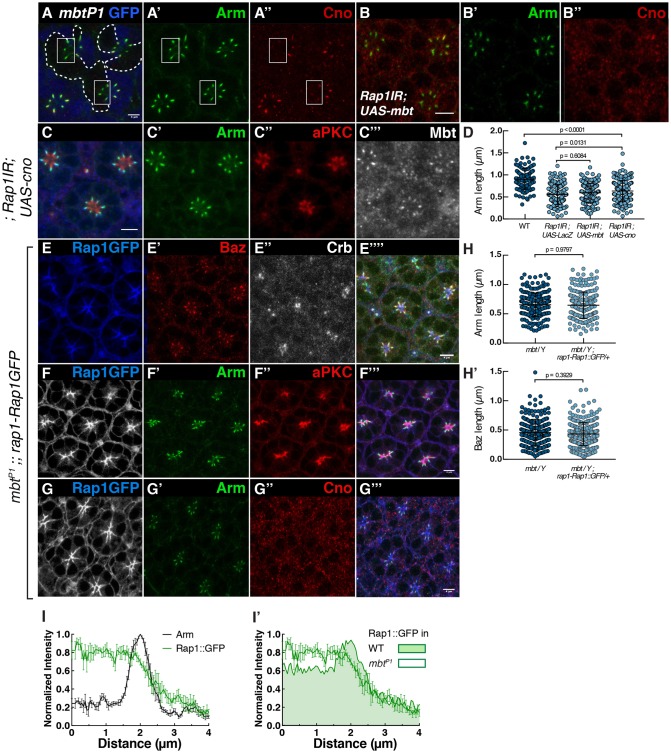
**Mbt is required for the accumulation of Cno and enrichment of Rap1 at the ZA.** (A) *mbt^P1^* mutant photoreceptors (lacking GFP, blue, the contour of which is denoted by the dashed line, A) and stained for Arm (A′) and Cno (A″). White boxes highlight ZAs within the *mbt^P1^* mutant tissue. (B) *Rap1IR* photoreceptors expressing Mbt and stained for Arm (B′) and Cno (B″). (C) *Rap1IR* photoreceptors expressing Cno and labeled for Arm (C′), aPKC (C″) and Mbt (C‴). (D) Quantification of the Arm domain length at the ZA in wild-type photoreceptors, and for *Rap1IR* photoreceptors co-expressing *UAS-LacZ*, *UAS-mbt* or *UAS-cno* driven by *NP-Gal4*^*2631*^. Results are mean±s.d. (*n*≥105 from 4 retinas). (E–G) *mbt^P1^* mutant photoreceptors expressing *rap1-Rap1::GFP* (E,F,G) stained for Baz (E′), Arm (F′,G′), Crb (E″), aPKC (F″) and Cno (G″). (H) Quantification of the length of the Arm (H) and Baz (H′) domains at the ZA in the *mbt^P1^* mutant and *mbt^P1^* mutants expressing *rap1-Rap1::GFP.* Results are mean±s.d. (*n*≥187 from 4 retinas). (I) Intensity profiles measured for Rap1::GFP and Arm along the apical-basal axis in *mbt^P1^* photoreceptors. (I′) Comparison of intensity profiles of Rap1::GFP measured in *mbt^P1^* photoreceptors compared to that of wild-type photoreceptors (shaded). Results are mean±s.e.m. (*n*≥6 cells from 3 retinas). Scale bars: 2 μm.

Our results indicate that Rap1 is required for the recruitment of Cno and Mbt at the photoreceptor ZA*.* Mbt is strongly decreased in *cnoIR* photoreceptors (Fig. 4E″,H″), which is compatible with Cno mediating Rap1 function in promoting Mbt accumulation at the ZA. Conversely, we find that Cno accumulation at the ZA depends on *mbt* (Fig. 5A–A″). Therefore, the localization of Cno and Mbt at the ZA are interlinked. To examine the functional relationship between Rap1, Cno and Mbt, and to test whether Cno and Mbt mediate Rap1 function during ZA morphogenesis, we asked whether expressing Mbt or Cno could ameliorate the *Rap1IR* ZA phenotype. We found even when expressing high levels of *mbt* (Fig. S5), the *Rap1IR* phenotype was not ameliorated (Fig. 5B,D). Similarly, expressing *cno* in *Rap1IR* cells did not restore Mbt accumulation to wild-type levels and did not ameliorate the length of the ZA (Fig. 5C,D).

Next, we assessed whether *Rap1* could mediate some of the *mbt* function by expressing the *rap1-Rap1::GFP* transgene in *mbt^P1^-*null mutant cells. *mbt^P1^* mutant cells are characterized by a decreased accumulation of Arm, Baz (Walther et al., 2016) (Fig. S6A,B) and Cno (Fig. 5A″) at their ZA. When expressing *rap1-Rap1::GFP* in *mbt^P1^* mutant cells (Fig. 5E–G), we did not measure any significant recovery in the length of the Arm (Fig. 5F′,G′,H) or Baz domains (Fig. 5E′,H′), when compared to that in *mbt^P1^* mutant cells, and Cno levels were not restored (Fig. 5G″). However, we noted that Rap1::GFP lacked the relative enrichment at the ZA normally detected in wild-type cells at this developmental stage (Figs 1F and 5I,I′).

One possibility is that Mbt might regulate the localization of Dzy, which in turn could shape that of Rap1. To test this possibility, we examined the localization of Dzy::GFP in *mbt^P1^* mutant photoreceptors and found it was much reduced when compared to wild type (Fig. S6C,D). It is therefore possible that Mbt influences Rap1 distribution along the apical-basal axis through Dzy.

### Dzy, Rap1, Cno synergize with Baz to promote AJ accumulation at the plasma membrane

**Fig. 6.**
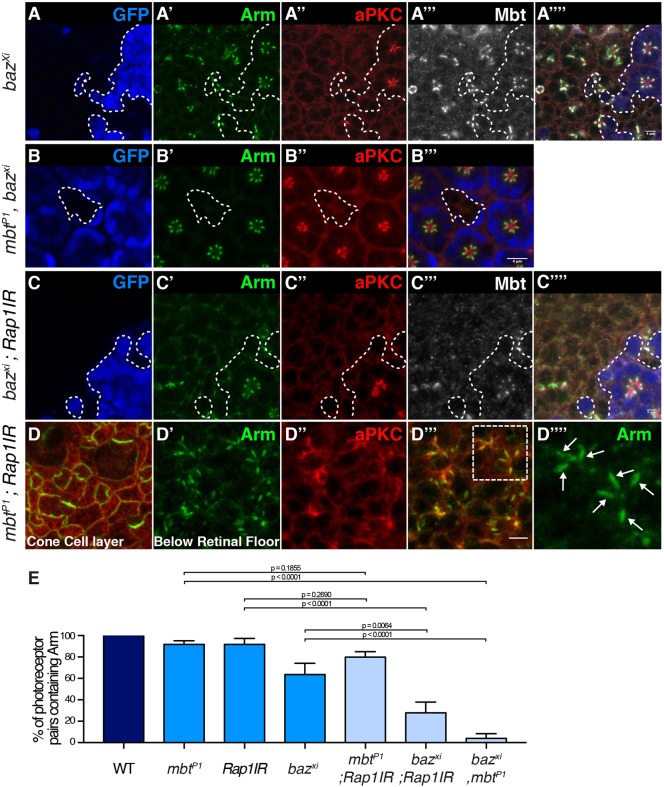
**Rap1, Cno and Mbt synergize with Baz to promote AJ accumulation at the plasma membrane.** (A–A‴′) *baz^xi106^* mutant cells (lacking GFP, blue, the contour of which is denoted by the dashed line, A) and stained for Arm (A′), aPKC (A″) and Mbt (A‴). (B–B‴) *mbt^P1^*, *baz^xi106^* double mutant cells (lacking GFP, blue, B) and stained for Arm (B′) and aPKC (B″). (C–C‴′) *baz^xi106^*, *Rap1IR* double mutant cells (lacking GFP, blue, C) and stained for Arm (C′), aPKC (C″) and Mbt (C‴). (D) Confocal section of the cone and pigment cells in an *mbt^P1^*; *Rap1IR* retina stained for Arm (green) and aPKC (red). (D′–D‴′) View of the delaminated photoreceptor proximal to D. (D′) Arm, (D″) aPKC, (D‴), merge (D″,D‴); a white-dashed rectangle highlights two ommatidia that are magnified in D‴′. The white arrows point to ZA domains between flanking photoreceptors. (E) Quantification of the percentage of pairs of photoreceptors sharing a lateral Arm domain in wild-type, *mbt^P1^, Rap1IR, baz ^xi106^,* double *mbt^P1^; Rap1IR,* double *baz ^xi106^; Rap1IR* and double *baz ^xi106^, mbt^P1^* cells. Results are mean±s.e.m. (*n*≥180 from 5 retinas). Scale bars: 4 μm.

In order to test whether the Rap1–Cno pathway mediates some of the Baz function in promoting AJ material accumulation at the plasma membrane, we made use of genetics to probe the relationship between *Rap1* and *baz*. First, we found that *Rap1* and *baz* genetically interact during eye development, as decreasing the expression of *baz* by using RNAi (*bazIR*), enhances the *Rap1IR* rough eye phenotype (Fig. S2A,B,E,F). Second, to assay whether Rap1 function during AJ morphogenesis relates to that of Baz we generated photoreceptors deficient for both *baz* (using the *baz^xi106^* allele) and *Rap1* (using the *NP-Gal4^2631^*-*Rap1IR* strain) (O'Keefe et al., 2009). As we have shown previously (Walther et al., 2016), AJ material, such as Arm, is detected at the plasma membrane in *baz^xi106^* and *mbt^P1^* single mutant cells (Fig. 6A′; Fig. S6B′). However, no AJ material is detected in *baz^xi106^ mbt^P1^* double-mutant cells (Fig. 6B) indicating that *baz* and *mbt* function through parallel pathways to promote AJ material accumulation at the plasma membrane. We found that expressing *Rap1IR* in *baz^xi106^* photoreceptors led to fewer cortical domains positive for Arm that are shared by flanking photoreceptors when compared to *baz^xi106^* and *Rap1IR* single mutant cells (Figs 6C″, 5E). This was accompanied by a loss of Mbt (Fig. 6C‴)*,* which is consistent with our observation that Rap1 is required for the accumulation of Mbt at the ZA (Fig. 2B″,E′). In contrast, AJ domains containing Arm are still present in double *mbt^P1^ Rap1IR* cells (Fig. 6D,E). Taken together, these data argue that while the respective functions of Rap1, Cno, Mbt and Baz converge during ZA morphogenesis, Rap1, Cno and Mbt function in parallel to Baz to promote AJ accumulation at the plasma membrane.

## DISCUSSION

In the pupal photoreceptor, ZA morphogenesis is orchestrated by a conserved protein network that includes Cdc42, Par6, aPKC, Baz, Crb and its binding partner Sdt, and Par1 (Berger et al., 2007; Hong et al., 2003; Izaddoost et al., 2002; Nam and Choi, 2003; Pellikka et al., 2002; Richard et al., 2006; Walther et al., 2016; Walther and Pichaud, 2010). In turn, AJ material is an essential part of the regulatory network that orchestrates polarity (Walther et al., 2016). We and others have previously shown that Mbt regulates pupal photoreceptor development by promoting ZA morphogenesis (Menzel et al., 2007; Walther et al., 2016). During this process Mbt contributes in preventing Baz from spreading to the lateral membrane, a regulation that we have found depends in part on the phosphorylation of Arm by Mbt at S561 and S688. We proposed that Mbt regulates photoreceptor polarity by promoting the retention of Baz at the developing ZA. Failure in ZA retention leads to Baz spreading to the lateral membrane where it is eliminated through Par1-mediated displacement. In these cells, failure to retain AJ material, including Baz, at the ZA leads to its shortening along the apical basal axis and can impact on the polarization program of the photoreceptor (Walther et al., 2016).

In the present study, we show that Mbt function is linked to that of Dzy, Rap1 and Cno. First, Cno and Mbt accumulation at the ZA is interdependent, reflecting a tight coupling between the Rap1 and Cno pathway and Mbt. Second, we find that Cno promotes Baz retention at the ZA, as *cnoIR* leads to shorter ZAs that can be depleted of Arm and Baz. This phenotype resembles that of *mbt* mutant cells and is also seen when overexpressing a version of Arm that cannot be phosphorylated by Mbt (Walther et al., 2016). These observations prompted us to test the hypothesis that Rap1, Cno and Mbt might function as part of a linear pathway promoting Baz retention at the ZA. In this pathway, we reasoned that Mbt could mediate Rap1 function through Arm phosphorylation. In testing this hypothesis, we found that this is not the case. Instead, the observation that expressing a version of Arm that mimics its constitutive phosphorylation by Mbt does not ameliorate the *cnoIR* phenotype suggests that Rap1, Cno, and Mbt converge in promoting Baz retention at the ZA, and cannot compensate for each other during this process. This conclusion is well supported by the finding that overexpressing *cno* in *mbt* mutant cells does not lead to an amelioration of the *mbt* phenotype. Third, we found that Mbt influences the distribution of Rap1 along the apical-basal axis of the cell in that Rap1::GFP no longer accumulates preferentially at the ZA*.* This correlates with a loss of Dzy::GFP at the plasma membrane, raising the possibility that Mbt might regulate Rap1 through Dzy. However, the *dzy* phenotype is milder than that seen with *Rap1* or *cno*, in that loss of *dzy* does not lead to cell delamination from the retina. This suggests that, as recently reported in the cellularizing embryo (Bonello et al., 2018; Schmidt et al., 2018), other GEFs regulate Rap1 during epithelial morphogenesis.

An interesting aspect of the *cnoIR* phenotype is the defects in apical accumulation of aPKC and Crb. These defects are not observed in the *dzy* mutant or *Rap1IR* cells, indicating that Cno might function independently of Rap1 during this process. However, we note that while we cannot detect Cno at the ZA of *cnoIR* cells, we still detect it in *Rap1IR* cells. We therefore hypothesize that residual Cno in *Rap1IR* cells supports optimum aPKC and Crb accumulation at the apical membrane. In our model, Dzy, Rap1 and Cno function as part of the same pathway, which includes a function in promoting optimum apical accumulation of Crb and aPKC. Baz is required for Par complex assembly and associated aPKC and Crb recruitment at photoreceptor apical membrane (Walther et al., 2016; Walther and Pichaud, 2010). We hypothesize that the defects in Crb and aPKC that we detect in *cnoIR* cells are linked to the failure in retaining Baz at the ZA, which leads to its elimination from the lateral membrane by Par1. More work will be required to understand how exactly AJ material and ZA retention of Baz influences apical membrane specification.

*Rap1* and *cno* have been shown to regulate apical-basal polarity in the cellularizing embryo. In this model system, Rap1 and Cno regulate the apical localization of Baz and Arm, which precedes the apical recruitment of Crb. In turn, Baz influences the localization of Cno (Choi et al., 2013). Our work indicates that similar complex regulations are at play in the pupal photoreceptor. However, unlike in the early embryo, AJ material (Arm) is absolutely required for Baz (and Par6–aPKC) accumulation or retention at the cell cortex in the developing pupal photoreceptor (Walther et al., 2016)*.* We therefore favor a model whereby Mbt, Rap1 and Cno influence ZA morphogenesis primarily through regulating the interface between E-Cad or Arm, Baz and the F-actin cytoskeleton. In this model, Mbt regulates this interface both through Arm phosphorylation and cofilin-dependent regulation of F-actin (Walther et al., 2016), and Cno contributes to this process, at least in part, through its ability to bind to F-actin.

To probe Rap1 and Cno function during photoreceptor ZA morphogenesis, we assessed the effect of decreasing Rap1 expression on E-Cad stability. Consistent with the notion that the function of *mbt* and *Rap1* are linked during ZA morphogenesis, we find that, as it is the case for Mbt (Walther et al., 2016), Rap1 is required to stabilize E-Cad::GFP at the photoreceptor ZA*.* However, the mobile fraction estimated for E-Cad is much higher in *Rap1IR* cells than in *mbt^P1^* null cells – evaluated at ∼70% for *Rap1IR* and 45% for *mbt^P1^* (Walther et al., 2016). Together with our finding that Mbt accumulation at the ZA is decreased in *Rap1IR* cells, our FRAP data are therefore compatible with Mbt mediating part of the function of Rap1 in promoting E-Cad stability. However, the much larger mobile fraction we estimate in the *Rap1IR* genotype when compared to *mbt^P1^* photoreceptors indicates that Rap1 must also regulate E-Cad stability independently of Mbt. The longer time scale for E-Cad::GFP to recover in *Rap1IR* cells when compared to *mbt^P1^* mutant cells is compatible with Rap1 functioning, in part, through promoting E-Cad delivery.

## MATERIALS AND METHODS

### Fly strains

The following fly strains were used: *rap1-Rap1::GFP* and *NP-Gal4^2631^, Rap1IR* (O'Keefe et al., 2009; BL #29434); *bazIR* (BL #39072), *cnoIR* (BL #33367) and *UAS-lacZ* (Bloomington Stock Center BL #3956); *dzy^Δ12^, FRT40A* (Huelsmann et al., 2006); *dzy-Dzy::GFP* (Boettner and Van Aelst, 200*7*); *ubi-Cad::GFP* (Oda and Tsukita, 2001); *mbt^P1^* and *UAS-Mbt* (Schneeberger and Raabe, 2003); *mbt^P1^, FRT19A;, mbt^P1^, baz^xi106^, FRT9.2;*, *;;UAS-Arm*, *;;UAS-ArmSA^mbt^* and *;;UAS-ArmSE^mbt^* (Walther et al., 2016); *w,baz^xi106^, FRT9.2* (Nusslein-Volhard et al., 1987); *FRT82B, cno^R2^* (Sawyer et al., 2009); *UAS-Cno* (Matsuo et al., 1997); *GMR-Gal4* (Freeman, 1996); and *NP-Gal4^2631^* (*Drosophila* Genomics Resource Center #104266) (Hayashi et al., 2002).

### Analysis of gene function

Clonal analysis of mutant alleles in the retina was performed using the standard FLP-FRT technique (Xu and Rubin, 1993) with appropriate *FRT*, *ubi-GFP* chromosomes used to generate negatively marked mutant tissue in combination with eyFLP (Newsome et al., 2000). Retina expressing RNAi in clones were generated by using the coinFLP system (Bosch et al., 2015). Clones of retinal tissue expressing RNAi against *Rap1* were generated both with and without UAS-dicer, while clones of retinal tissue expressing RNAi against *cno* were generated without UAS-dicer only. In order to mitigate the strong *Rap1* loss-of-function phenotype, *Rap1IR* animal were raised at 20°C and shifted to the appropriate temperature (25 or 29°C) at puparium formation. UAS transgenes were co-expressed with UAS-*Rap1IR* or UAS-*cnoIR* under the control of the *NP-Gal4^2631^* or *GMR-Gal4* drivers, respectively.

### Antibodies and immunological methods

Whole mount retinas at 40% after puparium formation (APF) were prepared as previously described (Walther and Pichaud, 2006). The following antibodies were used: rabbit anti-PKCζ (1:600, SAB4502380, Sigma), mouse anti-Arm (1:200, N27-A1, Developmental Studies Hybridoma Bank), rat anti-Baz, (1:1000, a gift from Andreas Wodarz, University of Cologne, Germany), rabbit anti-Cno, (1:200, a gift from Linda Van Aelst, Cold Spring Harbor Laboratory, New York, USA Boettner et al., 2003), rabbit anti-Baz (1:2000), rat anti-Crb (1:200), guinea pig anti-Mbt (1:200) (Walther et al., 2016), with the appropriate combination of mouse, guinea pig, rabbit and rat secondary antibodies conjugated to Dy405, Alexa Fluor 488, Cy3 or Cy5 as appropriate at 1:200 each (Jackson ImmunoResearch) or TRITC-conjugated Phalloidin (P1951, Sigma) at 2 μg/ml. Retinas were mounted in VectaShield™ with or without DAPI as appropriate, and imaging was performed by using a Leica SP5 confocal microscope. Images were edited with ImageJ and Adobe Photoshop 7.0.

### Western blot analysis

Pupal retina were dissected at 40% APF. For each genotype, ten retina were snap-frozen in PBS and SDS sample buffer was added to a final volume of 20 µl. Samples were analyzed by western blotting. Guinea pig anti-Mbt (Walther et al., 2016) and mouse anti-α-Tubulin antibodies (AA4.3, Developmental Studies Hybridoma Bank) (Walsh, 1984) were used for protein detection at concentrations of 1:1000 and 1:200, respectively.

### Data analysis

For length and pixel intensity measurements, a threshold was applied to define the ZA domain and a line was drawn along the apical-basal axis of the cell, running in the middle of the ZA to measure the length of the Arm, Baz and Mbt domains. Mean pixel intensity was measured by using the wand (tracing) tool in Fiji (Schindelin et al., 2012). In all cases, at least four independent mosaic retinas were used for each genotype. The intensity profiles of Rap1::GFP, dzy::GFP and Cno relative to Arm were measured in Fiji. A 1 µm line was drawn along the apical membrane and continued for 4 µm along the stalk membrane and ZA*.* For each profile, pixel intensities were subjected to unity-based normalization and adjusted such that the normalized maximum value of Arm was placed at 2 µm. Statistical analysis was performed in Prism 7.0. Data sets were tested for normality (D'Agostino and Pearson normality test) and *P*-values were calculated using a Student's *t*-test or the Mann–Whitney test as appropriate.

### Fluorescence recovery after photobleaching

FRAP analysis was performed as previously described (Walther et al., 2016). At 40% APF, the pupal cuticle was removed to expose the retina and the animal was mounted in Voltalef oil. Live imaging was performed on a Leica SP5 confocal microscope with a 63×1.4 NA oil immersion objective at the following settings: pixel resolution 512×512, speed 400 Hz, 10% 488 nm laser power at 20% argon laser intensity and 5× zoom. FRAP analysis of ubi*-*ECad::GFP was performed by marking the basal tip of the AJ with a 5 pixel diameter circlular region of interest (ROI) followed by photo-bleaching with a single pulse using 90% 488 nm laser power at 20% argon laser intensity. AJ recovery was recorded every 1.293 s with the previously mentioned settings for ∼1000 s. FRAP data were drift corrected in Fiji (Schindelin et al., 2012) using the StackReg plugin. Three different *z*-axis profiles were analyzed: (1) from the photo-bleached area; (2) from an equivalent area of a neighboring non-photo-bleached AJ; and (3) from an equivalent area of a background region. The data were normalized with easyFRAP. ECad::GFP data were fitted to a two-phase association curve in GraphPad Prism. The *P*-values were calculated with an unpaired two-tailed Student's *t*-test with Welch's correction.

### Scanning electron microscopy

Flies were fixed in 2% paraformaldehyde, 2% glutaraldehyde and 0.1 M cacodylate for 2 h and then dehydrated in ethanol, as previously described (Richardson and Pichaud, 2010). The samples were then critical-point dried and mounted on aluminum stubs before gold coating. Imaging was carried out on a JEOL Variable Pressure scanning electron microscope (SEM).

## Supplementary Material

Supplementary information
