# Supplementary material for: Rap1, Canoe and Mbt cooperate with Bazooka to promote zonula adherens assembly in the fly photoreceptor
Source: J Cell Sci. 2018 Mar 15;131(6):jcs207779. doi: 10.1242/jcs.207779 (PMC5897711; doi:10.1242/jcs.207779)
Supplement: Supplementary information [file joces-131-207779-s1.pdf]

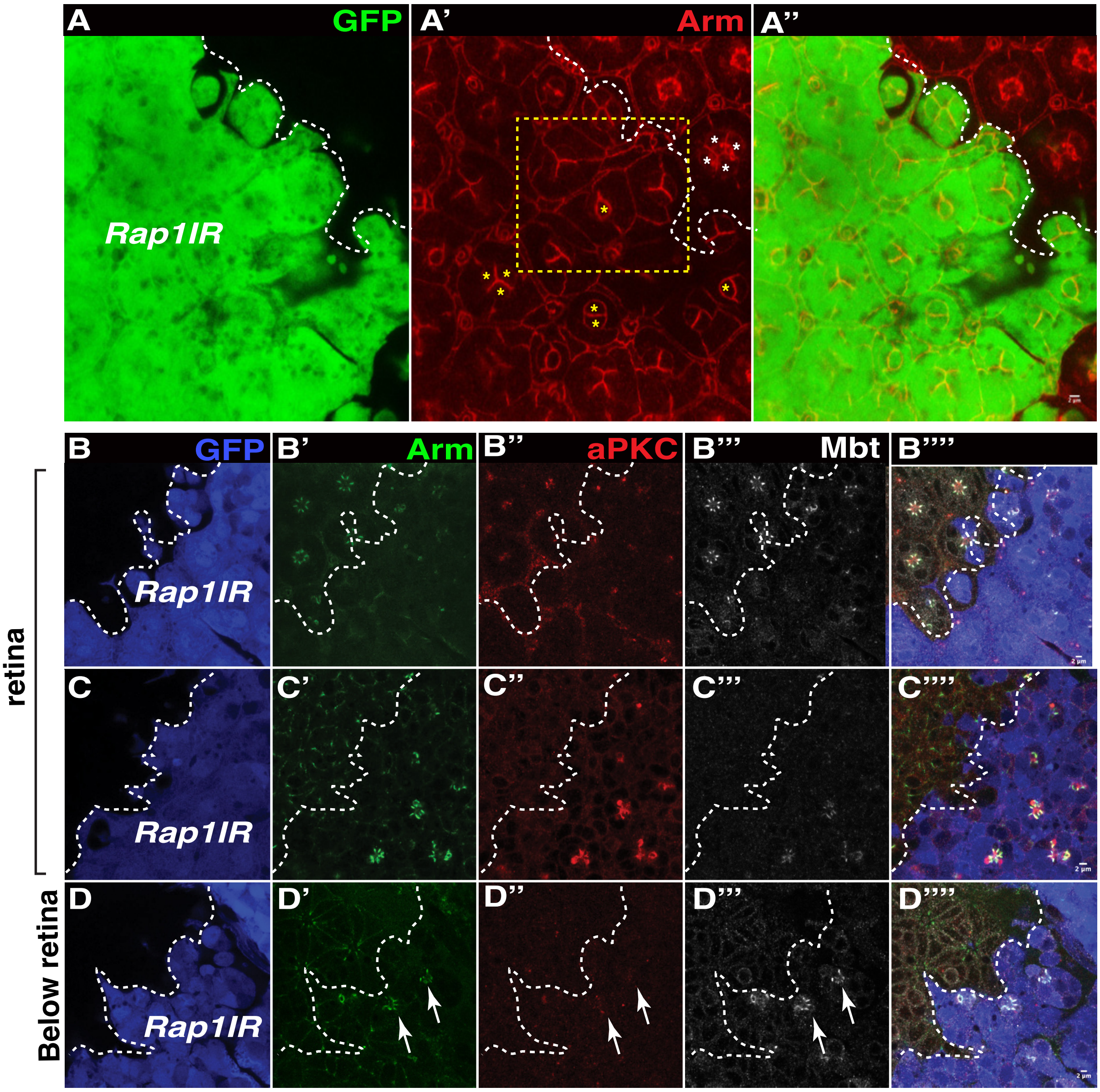

Supplementary Figure 1: Rap1 is required to preserve retinal tissue integrity. (A-A”) *Rap1IR* cells positively labeled by GFP (A) and stained for Arm (A’). Yellow stars label cone cells in the *Rap1IR* tissue. White stars label cone cells in one wild type ommatidium. Note the *Rap1IR* ommatidia lack cone cells. A yellow dashed box highlights *Rap1IR* ommatidia lacking interommatidial cells. (B-D) *Rap1IR* cells positively labeled by GFP (blue, (B, C and D)) and stained for Arm (B’, C’, D’), aPKC (B”, C”, D”) and Mbt (B’’’, C’’’, D’’’). Note that many *Rap1IR* photoreceptors delaminate below the floor of the retina, indicated by white arrows (D-D’’’’). Scale bars = 2µm.

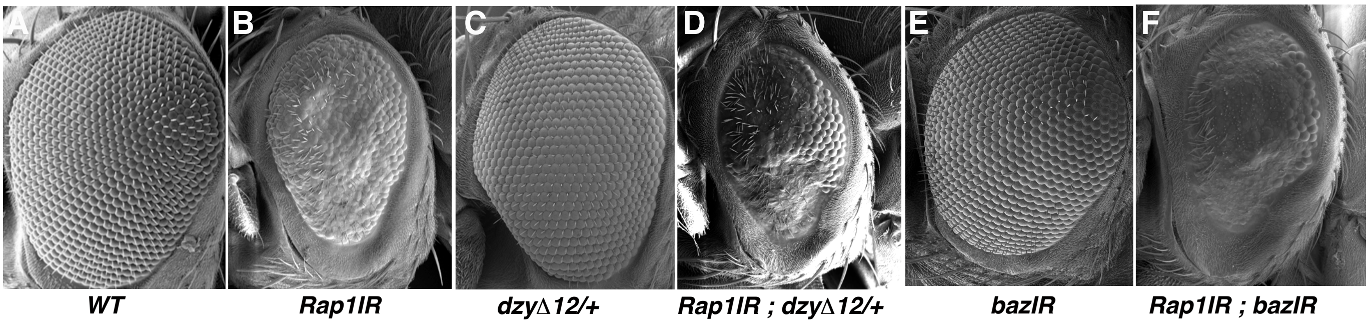

Supplementary Figure 2: Genetic modifiers of the *Rap1IR* rough eye phenotype. (A) SEM of a wild type eye, (B) *Rap1IR*, (C) Heterozygous $dzy^{\Delta 12}$ eye, (D) *Rap1IR* combined with $dzy^{\Delta 12}$ / +, (E) *bazIR,* (F) *bazIR* combined with *Rap1IR.*

Journal of Cell Science • Supplementary information

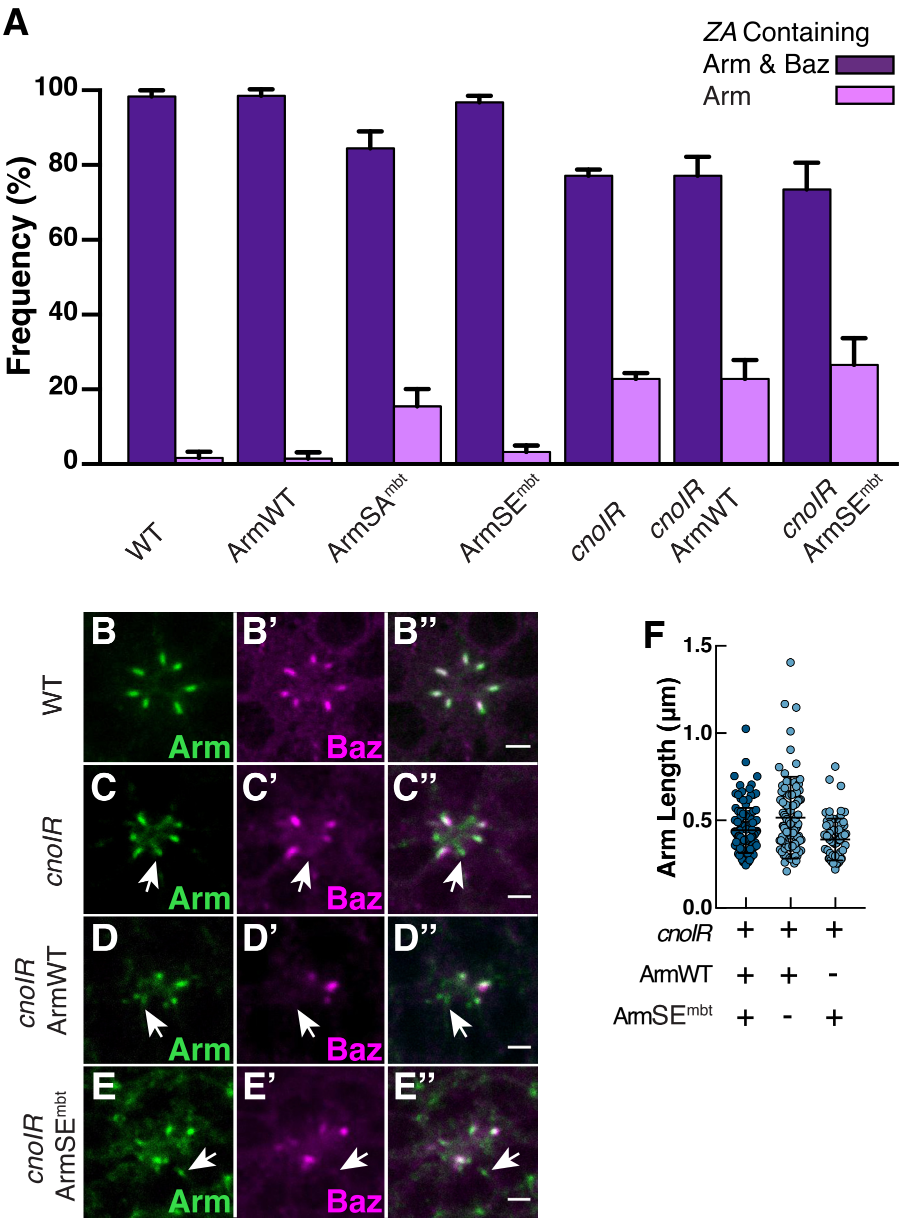

Supplementary Figure 3: Expression of ArmSE^{mbt} fails to rescue junction length and Baz retention in *cnoIR* photoreceptors

(A) Quantification of the percentage of photoreceptor *ZA* that contain both Arm and Baz (dark purple) or containing Arm but depleted for Baz (light purple). (B-E'') WT (B-B''), *cnoIR* (C-C''), *cnoIR* co-expressing ArmWT (D-D'') or *cnoIR* co-expressing ArmSE^{mbt} (E-E'') retina, stained for Arm (B, C, D, E) and Baz (B', C', D' and E'). White arrows indicate *ZA* that contain Arm but are depleted for Baz. Scale bars = 2µm. (F) Quantification of Arm domain length at the *ZA* in *cnoIR* photoreceptors and *cnoIR* photoreceptors expressing ArmWT or ArmSE^{mbt}.

Journal of Cell Science • Supplementary information

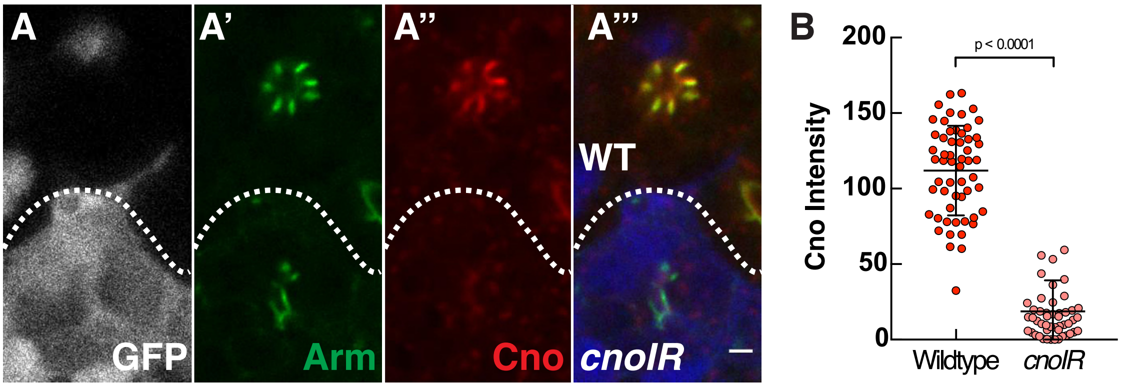

Supplementary Figure 4: *cnoIR* abolishes Cno expression

(A-A''') *cnoIR* cells positively labeled by GFP (A) and stained for Arm (A') and Cno (A''). Scale bars = 2µm. (B) Quantification of residual Cno intensity within the *ZA,* measured along the Arm domain, in *cnoIR* photoreceptors.

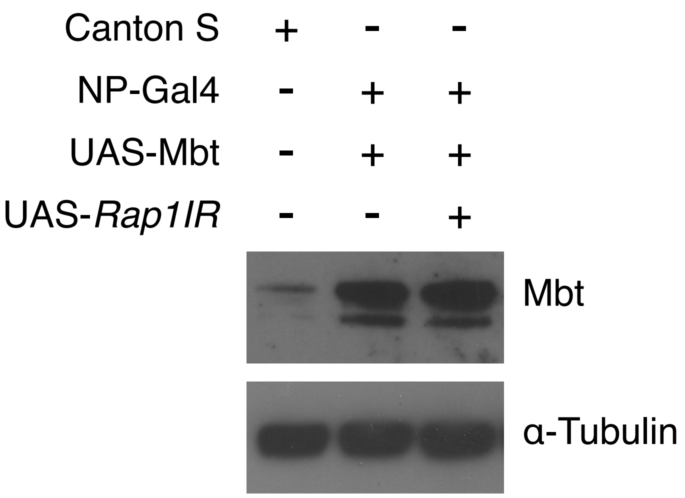

Supplementary Figure 5: Expression levels of Mbt in *Rap1IR* retinas

Western blot performed on retinal protein extracts, dissected at 40% after puparium formation.

Journal of Cell Science • Supplementary information

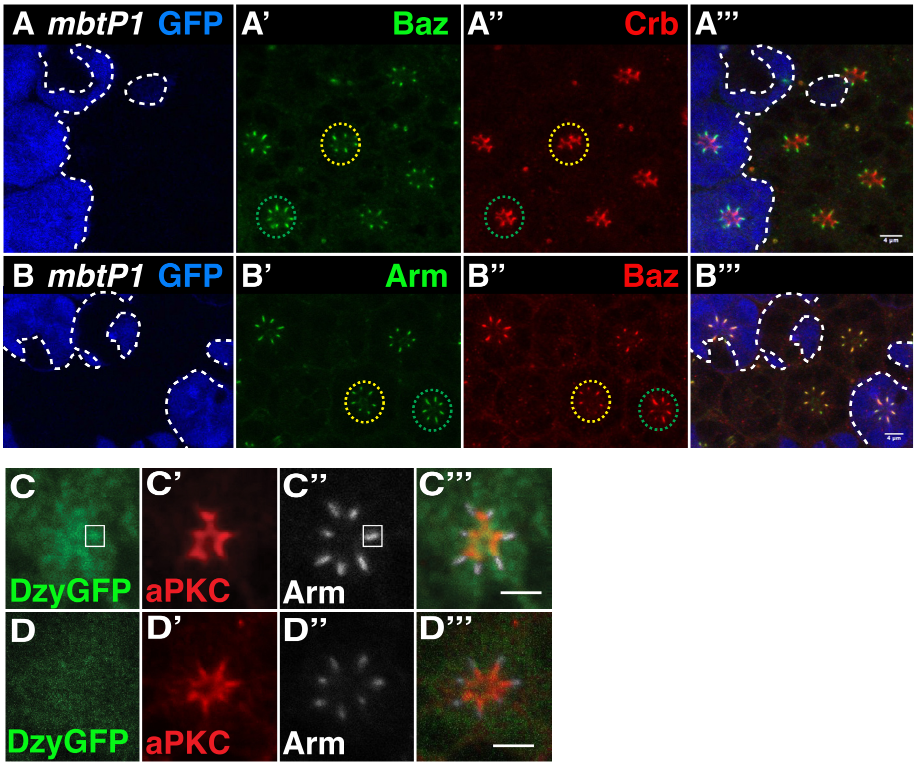

Supplementary Figure 6: Mbt Regulates the accumulation of Arm, Baz and Dzy at the *ZA*. (A-B) *mbt*^{P1} mutant cells (lacking of GFP, blue, (A and B)), stained for Baz (A' and B''), Arm (B') and Crb (A''). (C-C''') Dzy::GFP distribution in wild type photoreceptors (C), stained for aPKC (C') and Arm (C''). (D-D''') Dzy::GFP distribution in *mbt*^{P1} mutant photoreceptors (D), stained for aPKC (D') and Arm (D''). Scale bars = 2µm.
